# The Book World of Medicine and Science

**Published:** 1902-09-27

**Authors:** 


### The Book World of Medicine and Science.

Small-pox: An Inquiry into its Real Nature, etc. By C. Godfrey Gümpel. (London: Swan, Sonnenschein and Co., Limited. 1902. Pp. 38. Paper. Price 6d.)

This little book is, we believe, without exception, the most extraordinary farrago ever written about small-pox and vaccination. The author, Mr. Godfrey Gümpel, to whom all credit must be given for sincerity at least, speaks of it as "an attempt to submit the symptoms of the disease, and the factors which may cause it, to a closer analysis, and thus to guide us to the adoption of a probable and promising means by which to check the disorder in its first origins." No attempt could be more praiseworthy in its aspiration; few attempts can have failed so lamentably in the execution. In place of analysis or argument which could be combatted with logical weapons of precision we are presented with a series of positive misstatements and misconceptions that have no logical connection or scientific form. "Powerful factors," we are told, "such as the temporary state of the atmosphere, or the local electrical condition of the earth's surface, are periodically at work in the causation of small-pox epidemics." This statement is unsupported by any evidence from Mr. Gümpel, who makes, *àpropos de bottes*, the cryptic remark "that the electrical potential of the air we breathe takes no heed of medical assertions as to the prophylactic power of cow-pox." Mr. Gümpel recollects that in 1870–1871 there were in the autumn magnificent displays of the Aurora Borealis, and that about this time there was an epidemic of small-pox. He believes therefore that there is a close connection between the appearance of the Northern Lights and small-pox epidemics; but he does not state that any displays of these phenomena preceded the present one. The practical conclusion to which Mr. Gümpel comes is apparently that the action of the Northern Lights on the liver is best counteracted by the use of salt-solution as a beverage, and he appeals to the public to support him in inducing the Local Government Board, and the Metropolitan Asylums Board, to "adopt active steps to carry into effect" the administration of this "prophylatic remedy."

Hygiene and Public Health: A Manual of Personal and Public Health. By Arthur Newsholme, M.D., F.R.C.P., Lond. (London: Geo. Gill and Sons. New Edition. 1902. Price 4s. 6d.)

On opening this book we are confronted with the statement that "the writing of a preface is perhaps superfluous for a book which has had a large and steady sale for nearly twenty years," and we cannot but feel that the same might also be justly said regarding the writing of a review. At the same time as one hardly need remark the book as it stands before us is very different from what it was, a large amount of new matter having been introduced, much having been rewritten, and the whole of the subject matter having been brought well up to date. The date to which the information is brought up may be judged of by noting that the relation of the anopheles to malaria, of rats to plague, and of mosquitos to yellow fever are mentioned, and that Koch's statement, made in 1901, in regard to the non-identity of the bacilli of human and bovine tuberculosis is discussed. In a sense the book suffers a little, as all books do in which an attempt is made to render technical subjects comprehensible to non-technical readers, from the fact that it is not exclusively addressed either to the medical or the non-medical reader. The result is that, here and there, matters with which the medical student is sure to be familiar are treated in a somewhat elementary fashion for the edification of the outsider. This, however, is but a small matter, and as a general treatise on hygiene, well suited for the average medical student as well as for that large class of sanitary inspectors who wish to obtain a complete survey of the subject with one part of which they are engaged, we have every confidence in recommending this book. It is very comprehensive, taking in a very wide range of subjects, is accurate, it is readable, and is well illustrated.

Health, Speech, and Song: A Practical Guide to Voice Production. By Jutta Bell-Ranske. (London: Swan, Sonnenschein and Co. 1902. Price 4s. 6d. net.)

In this book there is a good deal that is interesting, and the leading ideas which the author endeavours to express appear to be true. Unfortunately there is also a great deal that is nonsensical. The author professes to base everything upon science. "An artistic temperament," we are told, "is more often than not a hindrance during the elementary stages of study, for no dramatic talent can be given freedom, or poetic temperament wings, until the agencies which have to be employed are got under control." We are further told that "the vocal organ is an instrument, and that its mechanism must be understood before we can use the voice with economy," and this is really the root idea of the book. Now in both these statements, so far as they are germane to the subject, there is a fallacy, for it has to be admitted that few orators or actors know much about the physiology of the instrument upon which they play with such effect, while a very slight acquaintance with current methods of voice production is sufficient to show how very little knowledge, or even unanimity in ignorance there is amongst the professors of that art. Nor do we think that the acquaintance with the subject displayed by the author of this book is such as to inspire great confidence in her power to excite her predecessors. We are told that "we have four different kinds of air in our lungs," one of which is "the tidal air, which is the air over and above that which is used in gentle breathing;" further on, that "the contraction of the diaphragm flattens the abdomen and invigorates all the various muscles that influence the liver and kidneys;" and still, again, that "when the abdomen is relaxed, the diaphragm arches up and forces the ribs in." Exactness of knowledge and precision of expression are essential in a teacher, and in one or another of these the author of this book certainly fails.

